# Supplementary material for: Mining logical circuits in fungi
Source: Sci Rep. 2022 Sep 23;12:15930. doi: 10.1038/s41598-022-20080-3 (PMC9508188; doi:10.1038/s41598-022-20080-3)
Supplement: Supplementary file 1 — Supplementary Information. [file 41598_2022_20080_MOESM1_ESM.pdf]

Supplementary materials
Mining logical circuits in fungi

Nic Roberts, Andrew Adamatzky

Unconventional Computing Laboratory, UWE, Bristol, UK

4-input-1-output logical functions discovered in experiments with mycelium bound composites.

$(A\overline{D}) + (C\overline{B}) + (D\overline{A}) + (\overline{AB}) + (BD\overline{C})$
$(A\overline{D}) + (D\overline{A}) + (BD\overline{C})$
$(AD\overline{C}) + (BD\overline{A}) + (AC\overline{BD})$
$(BD) + (AB\overline{C}) + (AC\overline{B})$
$(A\overline{D}) + (BD\overline{A})$
$(BC) + (BD) + (CD) + (A\overline{D}) + (D\overline{A})$
$(AB\overline{C}) + (ACD\overline{B})$
$(AD\overline{B}) + (D\overline{BC}) + (BCD\overline{A}) + (AB\overline{CD})$
$(B\overline{D}) + (D\overline{A}) + (AC\overline{D})$
$A + B + \overline{D}$
$(BC) + (BD) + (ACD) + (A\overline{CD})$
$(A\overline{D}) + (CD\overline{A})$
$(AD\overline{B}) + (A\overline{CD}) + (D\overline{BC}) + (BCD\overline{A})$
$(BC\overline{A}) + (ABD\overline{C}) + (ACD\overline{B}) + (A\overline{BCD})$
$AD\overline{C}$
$(BC) + (CD) + (A\overline{D}) + (D\overline{A})$
$\overline{C} + (A\overline{B}) + (B\overline{A}) + (D\overline{A})$
$(AB\overline{C}) + (AD\overline{B}) + (BD\overline{A}) + (CD\overline{A})$
$(AB\overline{C}) + (AC\overline{D})$
$(ABC\overline{D}) + (BCD\overline{A}) + (A\overline{BCD})$
$(AC\overline{B}) + (BD\overline{A}) + (A\overline{BD})$
$(B\overline{A}) + (C\overline{A}) + (D\overline{B}) + (BC\overline{D}) + (A\overline{BC})$
$(ABD) + (ACD)$
$(AB\overline{C}) + (AC\overline{B}) + (BC\overline{D}) + (BD\overline{C}) + (CD\overline{B})$

Preprint submitted to Journal *September 12, 2022*

$AB D\overline{C}$

$(A\overline{B}) + (B\overline{A}) + (D\overline{A}) + (BC\overline{D})$

$(A\overline{D}) + (AB\overline{C}) + (AC\overline{B}) + (BC\overline{A}) + (BC\overline{D}) + (BD\overline{A}) + (BD\overline{C}) + (D\overline{AC}) +$
$(\overline{ABC}) + (\overline{BCD})$

$(AB\overline{C}) + (AB\overline{D}) + (AC\overline{B}) + (AC\overline{D}) + (AD\overline{B}) + (AD\overline{C}) + (BD\overline{A}) + (BD\overline{C}) +$
$(CD\overline{A}) + (CD\overline{B})$

$(AB\overline{D}) + (A\overline{CD}) + (ACD\overline{B})$

$(B\overline{A}) + (C\overline{A}) + (D\overline{A}) + (BC\overline{D}) + (CD\overline{B})$

$(D\overline{A}) + (D\overline{B}) + (D\overline{C}) + (AB\overline{C}) + (AB\overline{D}) + (AC\overline{B}) + (AC\overline{D}) + (BC\overline{A}) + (BC\overline{D})$

$(B\overline{A}) + (B\overline{C}) + (B\overline{D}) + (A\overline{CD}) + (C\overline{AD}) + (D\overline{AC}) + (ACD\overline{B})$

$(A\overline{BCD}) + (D\overline{ABC})$

$(A\overline{D}) + (D\overline{A}) + (D\overline{B}) + (D\overline{C})$

$(D\overline{A}) + (D\overline{C}) + (BC\overline{A})$

$(A\overline{D}) + (D\overline{A}) + (D\overline{BC})$

$(A\overline{D}) + (D\overline{A}) + (AB\overline{C}) + (AC\overline{B}) + (BC\overline{A}) + (BC\overline{D}) + (BD\overline{C}) + (CD\overline{B}) +$
$(\overline{ABC}) + (\overline{BCD})$

$(AB\overline{D}) + (AC\overline{B}) + (CD\overline{A}) + (D\overline{AB})$

$(A\overline{D}) + (BC\overline{A}) + (D\overline{ABC})$

$(BC\overline{A}) + (BD\overline{C}) + (ACD\overline{B}) + (\overline{BCD})$

$(A\overline{D}) + (C\overline{B}) + (D\overline{A}) + (D\overline{C})$

$(AD\overline{C}) + (BD\overline{A}) + (CD\overline{A})$

$(A\overline{C}) + (BC\overline{D}) + (BD\overline{A}) + (CD\overline{B})$

$(A\overline{B}) + (A\overline{C}) + (D\overline{A})$

$(A\overline{B}) + (C\overline{A}) + (D\overline{C})$

$(D\overline{A}) + (D\overline{B}) + (AB\overline{C}) + (BC\overline{A})$

$(AB\overline{D}) + (AC\overline{D})$

$(ABC\overline{D}) + (ACD\overline{B}) + (BCD\overline{A})$

$(BC) + (A\overline{D}) + (D\overline{A}) + (D\overline{B})$

$(AC\overline{B}) + (BC\overline{A}) + (BD\overline{C}) + (A\overline{CD})$

$(AB\overline{C}) + (A\overline{BD}) + (BCD\overline{A})$

$(A\overline{D}) + (BC\overline{D}) + (D\overline{AB}) + (D\overline{AC})$

$\overline{A} + \overline{B} + (C\overline{D}) + (D\overline{C})$

$(B\overline{AC}) + (B\overline{AD}) + (B\overline{CD})$

$(A\overline{CD}) + (ACD\overline{B}) + (BC\overline{AD})$

$(AB\overline{C}) + (ACD\overline{B}) + (BC\overline{AD})$

$(AB\overline{C}) + (AC\overline{B}) + (AD\overline{B}) + (BC\overline{A}) + (\overline{ABCD})$

$(B\overline{A}) + (C\overline{AD}) + (ACD\overline{B})$

$(A\overline{CD}) + (ACD\overline{B})$

$(AB\overline{C}) + (ACD\overline{B}) + (BCD\overline{A})$
$(AD\overline{C}) + (BD\overline{A})$
$\overline{A} + \overline{D} + (B\overline{C}) + (C\overline{B})$
$(D\overline{A}) + (D\overline{B}) + (D\overline{C}) + (AC\overline{B}) + (AC\overline{D}) + (BC\overline{A}) + (BC\overline{D})$
$(A\overline{D}) + (D\overline{A}) + (BC\overline{A}) + (BD\overline{C}) + (CD\overline{B})$
$\overline{C} + (BD) + (A\overline{B}) + (B\overline{A})$
$(BC) + (BD) + (CD) + (D\overline{A}) + (A\overline{CD})$
$(A\overline{D}) + (D\overline{A}) + (D\overline{C})$
$(A\overline{D}) + (B\overline{C}) + (C\overline{A}) + (C\overline{B}) + (D\overline{A})$
$(A\overline{C}) + (D\overline{C}) + (AD\overline{B}) + (BC\overline{D})$
$B + \overline{A} + \overline{D}$
$(AC\overline{D}) + (BD\overline{A})$
$\overline{A} + \overline{B} + \overline{D}$
$(A\overline{D}) + (B\overline{A}) + (B\overline{C}) + (CD\overline{B})$
$(D\overline{A}) + (D\overline{B}) + (AB\overline{C})$
$(A\overline{D}) + (B\overline{A}) + (B\overline{C}) + (B\overline{D}) + (D\overline{A}) + (\overline{AC}) + (\overline{CD}) + (AC\overline{B}) + (CD\overline{B})$
$(D\overline{A}) + (D\overline{C}) + (AC\overline{B}) + (BC\overline{A})$
$(ACD\overline{B}) + (AB\overline{CD})$
$(ABD\overline{C}) + (A\overline{BCD}) + (D\overline{ABC})$
$(A\overline{D}) + (D\overline{A}) + (D\overline{B}) + (D\overline{C}) + (\overline{BC})$
$(A\overline{B}) + (A\overline{C}) + (BC\overline{A}) + (BD\overline{A}) + (\overline{BCD})$
$(A\overline{BD}) + (A\overline{CD})$
$(A\overline{B}) + (A\overline{C}) + (A\overline{D}) + (BC\overline{A}) + (BC\overline{D}) + (BD\overline{A}) + (BD\overline{C}) + (CD\overline{A}) +$
$(CD\overline{B}) + (\overline{BCD})$
$(B\overline{D}) + (AB\overline{C}) + (BC\overline{A}) + (A\overline{CD}) + (ACD\overline{B})$
$(BCD) + (B\overline{CD}) + (AC\overline{BD}) + (AD\overline{BC})$
$(BC\overline{A}) + (BD\overline{C}) + (ACD\overline{B}) + (A\overline{BCD})$
$(A\overline{D}) + (B\overline{A}) + (B\overline{D}) + (C\overline{A}) + (C\overline{D}) + (D\overline{A}) + (A\overline{BC}) + (D\overline{BC})$
$(A\overline{B}) + (AD\overline{C}) + (BC\overline{A}) + (\overline{BCD})$
$(A\overline{D}) + (AC\overline{B}) + (BC\overline{A}) + (BD\overline{A})$
$(AC\overline{B}) + (AC\overline{D}) + (A\overline{BD}) + (BCD\overline{A})$
$(B\overline{D}) + (C\overline{D}) + (D\overline{A}) + (D\overline{BC})$
$(B\overline{C}) + (B\overline{D}) + (C\overline{B}) + (C\overline{D}) + (D\overline{A}) + (D\overline{B}) + (D\overline{C})$
$(A\overline{B}) + (A\overline{D}) + (B\overline{A}) + (B\overline{D}) + (C\overline{A}) + (D\overline{A}) + (D\overline{B})$
$(A\overline{D}) + (AB\overline{C}) + (AC\overline{B}) + (BC\overline{A}) + (\overline{BCD})$
$A + B + D + \overline{C}$
$(AC\overline{B}) + (BCD\overline{A})$
$(AB\overline{C}) + (AC\overline{B}) + (A\overline{BD})$

$(AB) + (CD\overline{B}) + (A\overline{CD}) + (D\overline{AC})$

$(A\overline{D}) + (BC\overline{A}) + (BD\overline{A}) + (CD\overline{B})$

$(AB\overline{D}) + (AC\overline{B}) + (BCD\overline{A})$

$(BC\overline{A}) + (CD\overline{A}) + (A\overline{BD}) + (A\overline{CD})$

$(B\overline{D}) + (AB\overline{C}) + (BC\overline{A}) + (A\overline{CD}) + (ACD\overline{B}) + (D\overline{ABC})$

$\overline{D} + (A\overline{B}) + (A\overline{C}) + (B\overline{A}) + (B\overline{C}) + (C\overline{A}) + (C\overline{B})$

$(AC\overline{B}) + (AD\overline{C}) + (BCD\overline{A})$

$(AB\overline{C}) + (AB\overline{D}) + (BC\overline{A}) + (BC\overline{D}) + (BD\overline{A}) + (BD\overline{C}) + (CD\overline{B})$

$(A\overline{CD}) + (BCD\overline{A})$

$(AD\overline{B}) + (BCD\overline{A})$

$(A\overline{BC}) + (A\overline{CD}) + (BC\overline{AD})$

$(B\overline{AC}) + (C\overline{BD}) + (D\overline{AB})$

$(A\overline{D}) + (D\overline{A}) + (BC\overline{A}) + (CD\overline{B}) + (\overline{ABC})$

$(A\overline{D}) + (D\overline{A}) + (D\overline{C}) + (\overline{BC}) + (BC\overline{A})$

$(B\overline{A}) + (B\overline{D}) + (A\overline{BC}) + (C\overline{AD}) + (D\overline{BC})$

$(AB\overline{C}) + (AB\overline{D}) + (A\overline{CD}) + (ACD\overline{B}) + (BCD\overline{A})$

$(C\overline{D}) + (D\overline{B}) + (D\overline{C}) + (AB\overline{C})$

$(C\overline{B}) + (D\overline{A}) + (\overline{BD}) + (AB\overline{C})$

$(AC\overline{B}) + (AC\overline{D}) + (BCD\overline{A})$

$(B\overline{D}) + (C\overline{A}) + (C\overline{B}) + (C\overline{D}) + (\overline{AD}) + (AB\overline{C}) + (AD\overline{B}) + (AD\overline{C})$

$(AC\overline{B}) + (A\overline{CD}) + (BC\overline{AD})$

$(ABD\overline{C}) + (ACD\overline{B}) + (A\overline{BCD}) + (D\overline{ABC})$

$(A\overline{D}) + (BCD) + (BD\overline{A}) + (C\overline{BD})$

$(A\overline{D}) + (AB\overline{C}) + (BCD\overline{A})$

$(BD\overline{C}) + (A\overline{BD})$

$(A\overline{B}) + (A\overline{C}) + (BC\overline{A}) + (\overline{BCD})$

$(B\overline{A}) + (B\overline{C}) + (C\overline{D}) + (AC\overline{B}) + (D\overline{AC})$

$(ABD\overline{C}) + (ACD\overline{B}) + (BCD\overline{A})$

$(AB\overline{D}) + (C\overline{BD})$

$(D\overline{A}) + (D\overline{B}) + (D\overline{C}) + (AB\overline{C}) + (AC\overline{B}) + (BC\overline{A})$

$(C\overline{BD}) + (AB\overline{CD})$

$(B\overline{C}) + (D\overline{A}) + (D\overline{B}) + (AB\overline{D}) + (C\overline{AB})$

$(B\overline{C}) + (AB\overline{D}) + (AC\overline{B}) + (AC\overline{D}) + (AD\overline{B}) + (AD\overline{C}) + (BD\overline{A}) + (CD\overline{A}) +$
$(CD\overline{B}) + (\overline{ACD})$

$(D\overline{A}) + (D\overline{B}) + (D\overline{C}) + (AC\overline{D})$

$(AB\overline{C}) + (AB\overline{D}) + (CD\overline{B}) + (A\overline{CD}) + (D\overline{AB})$

$(BD) + (AB\overline{C}) + (AC\overline{B}) + (BC\overline{A})$

$(AB) + (AC) + (A\overline{D}) + (BC\overline{D})$

$(C\overline{D}) + (D\overline{C}) + (AB\overline{C}) + (AD\overline{B})$
$(C\overline{B}) + (D\overline{A}) + (\overline{AB}) + (A\overline{CD})$
$(AB\overline{C}) + (AC\overline{D}) + (AD\overline{C}) + (BD\overline{A})$
$(A\overline{B}) + (A\overline{D}) + (B\overline{A}) + (B\overline{D}) + (D\overline{A}) + (D\overline{B})$
$(D\overline{A}) + (D\overline{C}) + (AC\overline{B})$
$(A\overline{D}) + (B\overline{A}) + (B\overline{C}) + (C\overline{D}) + (AC\overline{B})$
$(BCD) + (AB\overline{C}) + (AC\overline{B})$
$(BC\overline{A}) + (A\overline{BD}) + (A\overline{CD})$
$(AB\overline{C}) + (AB\overline{D}) + (CD\overline{A}) + (CD\overline{B})$
$(AC\overline{B}) + (C\overline{BD}) + (AB\overline{CD})$
$(B\overline{A}) + (C\overline{A}) + (B\overline{CD}) + (C\overline{BD}) + (AD\overline{BC})$
$(A\overline{B}) + (A\overline{C}) + (B\overline{A}) + (B\overline{C}) + (C\overline{A}) + (C\overline{B}) + (D\overline{A})$
$(A\overline{D}) + (D\overline{A}) + (D\overline{B}) + (\overline{BC}) + (BC\overline{A})$
$(ABD) + (BCD) + (A\overline{BCD})$
$(ABD) + (ACD) + (BCD) + (\overline{BCD})$
$(ABC\overline{D}) + (ACD\overline{B}) + (BCD\overline{A}) + (A\overline{BCD}) + (D\overline{ABC})$
$(AB\overline{C}) + (AC\overline{B}) + (A\overline{BD}) + (BCD\overline{A})$
$(AB\overline{C}) + (AB\overline{D}) + (CD\overline{B}) + (A\overline{CD})$
$ABCD$
$(AC\overline{B}) + (BC\overline{A}) + (A\overline{CD}) + (B\overline{CD}) + (C\overline{AD})$
$(ABD\overline{C}) + (ACD\overline{B})$
$(A\overline{B}) + (A\overline{C}) + (\overline{BC}) + (BD\overline{A})$
$(A\overline{C}) + (B\overline{D}) + (D\overline{B}) + (D\overline{C})$
$(A\overline{B}) + (B\overline{D})$
$(AB\overline{C}) + (AC\overline{D}) + (AD\overline{C}) + (BD\overline{A}) + (CD\overline{A})$
$(A\overline{CD}) + (ACD\overline{B}) + (BCD\overline{A})$
$ACD\overline{B}$
$(B\overline{A}) + (C\overline{A}) + (D\overline{B}) + (A\overline{BC})$
$(AC\overline{B}) + (A\overline{CD}) + (BCD\overline{A})$
$A + D + (BC) + (\overline{BC})$
$(A\overline{B}) + (A\overline{C}) + (\overline{CD}) + (B\overline{AD})$
$(D\overline{A}) + (D\overline{B}) + (D\overline{C}) + (AB\overline{C}) + (AB\overline{D}) + (AC\overline{B}) + (AC\overline{D})$
$A + D + (BC)$
$(A\overline{D}) + (B\overline{A}) + (D\overline{A})$
$(A\overline{CD}) + (BD\overline{AC})$
$(BC\overline{D}) + (BD\overline{A}) + (BD\overline{C}) + (CD\overline{B}) + (A\overline{CD})$
$(A\overline{B}) + (A\overline{C}) + (A\overline{D}) + (BC\overline{A})$
$(A\overline{D}) + (C\overline{A}) + (D\overline{A}) + (D\overline{B})$

ABD

$B+(AD)+(A\overline{C})$

$AC\overline{BD}$

$(AB\overline{C})+(AD\overline{B})+(BC\overline{AD})$

$BCD\overline{A}$

$(A\overline{D})+(AC\overline{B})+(BC\overline{A})+(BD\overline{C})+(D\overline{AC})$

$(B\overline{AC})+(B\overline{CD})+(C\overline{AD})$

$(A\overline{D})+(D\overline{AB})+(D\overline{AC})$

$(A\overline{CD})+(ACD\overline{B})+(BD\overline{AC})$

$(A\overline{D})+(AC\overline{B})+(BCD\overline{A})$

$(D\overline{A})+(D\overline{C})+(AC\overline{B})+(B\overline{AC})$

$A\overline{D}$

$(AB\overline{D})+(BCD\overline{A})$

$A+(BC)+(BD)+(\overline{BCD})$

$(BD\overline{A})+(AD\overline{BC})$

$(AC\overline{D})+(BD\overline{C})+(A\overline{BD})$

$(ABC\overline{D})+(A\overline{BCD})$

$(AC\overline{B})+(AD\overline{C})+(BD\overline{A})+(CD\overline{A})$

$(AB\overline{C})+(AB\overline{D})+(CD\overline{A})+(CD\overline{B})+(A\overline{CD})$

$(AB\overline{CD})+(BC\overline{AD})$

$(AC\overline{B})+(BC\overline{A})+(BD\overline{A})+(A\overline{CD})$

$(AC)+(CD)+(A\overline{D})+(B\overline{A})+(D\overline{A})+(\overline{AC})+(\overline{CD})$

$(AB\overline{D})+(A\overline{CD})$

$(A\overline{D})+(D\overline{A})+(CD\overline{B})$

$(B\overline{AC})+(C\overline{BD})+(D\overline{AC})$

$(A\overline{D})+(CD\overline{A})+(CD\overline{B})+(D\overline{AB})$

$(AB)+(AC)+(A\overline{D})+(B\overline{D})+(D\overline{AB})$

$(A\overline{B})+(A\overline{C})+(B\overline{D})+(\overline{BC})+(BC\overline{A})$

$(A\overline{D})+(BC\overline{A})+(D\overline{AB})$

$(AB\overline{D})+(A\overline{CD})+(ACD\overline{B})+(BCD\overline{A})+(D\overline{ABC})$

$(AB\overline{C})+(AB\overline{D})+(AC\overline{B})+(BD\overline{A})$

$\overline{C}+\overline{D}+(A\overline{B})+(B\overline{A})$

$(AB\overline{D})+(ACD\overline{B})+(BCD\overline{A})$

$(A\overline{B})+(B\overline{A})+(B\overline{C})+(D\overline{A})$

$(AB\overline{C})+(AC\overline{B})+(A\overline{BD})+(BC\overline{AD})$

$(B\overline{A})+(B\overline{C})+(B\overline{D})+(D\overline{A})+(D\overline{B})+(D\overline{C})+(AC\overline{B})+(AC\overline{D})$

$(B\overline{A})+(C\overline{D})+(D\overline{A})$

$(A\overline{D})+(AB\overline{C})+(AC\overline{B})+(BC\overline{D})$

$(A\overline{B}) + (A\overline{D}) + (BC\overline{D})$
$(B\overline{C}) + (AC\overline{D}) + (CD\overline{A}) + (CD\overline{B})$
$(BCD\overline{A}) + (AD\overline{BC})$
$(AC\overline{BD}) + (BC\overline{AD})$
$(AD) + (BD) + (BC\overline{A}) + (\overline{BCD})$
$(A\overline{B})+(A\overline{C})+(A\overline{D})+(BC\overline{A})+(BC\overline{D})+(BD\overline{A})+(BD\overline{C})+(CD\overline{A})+(CD\overline{B})$
$(CD\overline{B}) + (A\overline{CD}) + (D\overline{AC})$
$(A\overline{D}) + (D\overline{A}) + (D\overline{B}) + (BC\overline{A})$
$(AB) + (ACD) + (BCD) + (\overline{BCD})$
$(A\overline{D}) + (BD\overline{AC}) + (CD\overline{AB})$
$\overline{A} + (B\overline{C}) + (C\overline{B}) + (\overline{BD})$
$(AB\overline{C}) + (AC\overline{B}) + (\overline{ABCD})$
$(BCD) + (A\overline{BD}) + (A\overline{CD}) + (B\overline{AD}) + (B\overline{CD}) + (C\overline{AD}) + (C\overline{BD}) + (D\overline{BC})$
$(A\overline{B}) + (A\overline{C}) + (B\overline{D}) + (BC\overline{A})$
$(AC\overline{B}) + (CD\overline{A}) + (A\overline{CD}) + (D\overline{AB})$
$(A\overline{C}) + (AD\overline{B}) + (BC\overline{D}) + (BD\overline{C})$
$(D\overline{C}) + (\overline{BC}) + (AD\overline{B}) + (BD\overline{A})$
$(A\overline{B}) + (AD\overline{C}) + (BCD\overline{A}) + (\overline{BCD})$
$(A\overline{D}) + (BC\overline{A}) + (BD\overline{C}) + (CD\overline{B})$
$(A\overline{B}) + (A\overline{C}) + (B\overline{D}) + (\overline{CD}) + (BC\overline{A})$
$(A\overline{D}) + (C\overline{BD}) + (BCD\overline{A})$
$\overline{C} + (A\overline{B}) + (A\overline{D}) + (B\overline{A}) + (B\overline{D}) + (D\overline{A}) + (D\overline{B})$
$(A\overline{D}) + (B\overline{A}) + (B\overline{C}) + (D\overline{A})$
$(B\overline{C}) + (D\overline{C}) + (AB\overline{D}) + (AD\overline{B}) + (BD\overline{A}) + (C\overline{ABD})$
$B + C + \overline{A} + \overline{D}$
$(AB\overline{C}) + (BC\overline{A}) + (BC\overline{D}) + (ACD\overline{B}) + (\overline{BCD})$
$(D\overline{A}) + (AB\overline{C}) + (A\overline{BD})$
$A + B + C + D$
$(A\overline{B}) + (A\overline{CD}) + (BC\overline{AD})$
$(AC\overline{D}) + (BD\overline{A}) + (CD\overline{A})$
$(D\overline{B}) + (CD\overline{A}) + (A\overline{CD})$
$(A\overline{B}) + (B\overline{A}) + (BC\overline{D}) + (CD\overline{A})$
$\overline{A} + \overline{D} + (B\overline{C})$
$(AB\overline{C}) + (AC\overline{B}) + (AD\overline{B}) + (BC\overline{AD})$
$A + C + D + \overline{B}$
$(D\overline{A}) + (D\overline{B}) + (A\overline{CD}) + (C\overline{AB})$
$(CD) + (A\overline{D}) + (C\overline{B}) + (D\overline{A})$
$(A\overline{D}) + (BD\overline{C}) + (CD\overline{B}) + (\overline{BCD})$

$(D\overline{A}) + (D\overline{C}) + (AB\overline{C}) + (AC\overline{D}) + (BC\overline{A})$
$(A\overline{C}) + (B\overline{D}) + (C\overline{A}) + (D\overline{A}) + (D\overline{B})$
$(ABD\overline{C}) + (BCD\overline{A}) + (AC\overline{BD})$
$(AB\overline{C}) + (BC\overline{A}) + (BC\overline{D}) + (A\overline{CD}) + (ACD\overline{B})$
$(ABD\overline{C}) + (BCD\overline{A})$
$(B\overline{A}) + (B\overline{C}) + (AC\overline{B}) + (A\overline{BD})$
$(A\overline{C}) + (B\overline{D}) + (D\overline{A}) + (D\overline{B})$
$(A\overline{D}) + (BCD\overline{A})$
$(A\overline{D}) + (B\overline{A}) + (D\overline{A}) + (CD\overline{B})$
$(B\overline{A}) + (B\overline{C}) + (D\overline{C}) + (AC\overline{B}) + (C\overline{BD})$
$(ACD\overline{B}) + (AB\overline{CD}) + (BC\overline{AD})$
$(B\overline{A}) + (C\overline{A}) + (CD\overline{B})$
$(B\overline{C}) + (D\overline{A}) + (D\overline{B}) + (D\overline{C}) + (\overline{AC}) + (AB\overline{D}) + (AC\overline{B}) + (AC\overline{D})$
$(A\overline{BD}) + (A\overline{CD}) + (BCD\overline{A})$
$(ABC\overline{D}) + (ABD\overline{C}) + (ACD\overline{B}) + (BCD\overline{A})$
$(B\overline{A}) + (B\overline{D}) + (C\overline{A}) + (C\overline{D}) + (AD\overline{BC})$
$ABC\overline{D}$
$A + B + (\overline{CD})$
$(AB\overline{C}) + (AB\overline{D}) + (AC\overline{B}) + (BC\overline{A}) + (BC\overline{D}) + (BD\overline{A}) + (BD\overline{C})$
$(BCD) + (B\overline{CD}) + (C\overline{AD}) + (C\overline{BD}) + (D\overline{BC})$
$(AB\overline{C}) + (AC\overline{B}) + (AD\overline{B}) + (BC\overline{D})$
$(C\overline{BD}) + (D\overline{AB}) + (B\overline{ACD})$
$(D\overline{A}) + (D\overline{B}) + (AB\overline{C}) + (AC\overline{B})$
$(AD\overline{C}) + (BCD\overline{A}) + (AC\overline{BD})$
$(D\overline{B}) + (D\overline{C}) + (BC\overline{A}) + (A\overline{BC})$
$(AC\overline{B}) + (C\overline{BD}) + (BCD\overline{A}) + (AB\overline{CD})$
$(B\overline{A}) + (AD\overline{B}) + (B\overline{CD}) + (C\overline{AD})$
$\overline{A} + \overline{B} + \overline{C} + \overline{D}$
$(BD) + (A\overline{B}) + (BC\overline{A}) + (\overline{BCD})$
$(ABD\overline{C}) + (A\overline{BCD})$
$(A\overline{B}) + (A\overline{C}) + (A\overline{D}) + (D\overline{A}) + (D\overline{B}) + (D\overline{C}) + (BC\overline{A}) + (BC\overline{D})$
$(A\overline{D}) + (BD\overline{C}) + (CD\overline{B}) + (\overline{ABC})$
$(AC\overline{B}) + (A\overline{CD}) + (D\overline{ABC})$
$(AB\overline{D}) + (AD\overline{B}) + (AD\overline{C}) + (BCD\overline{A})$
$(B\overline{C}) + (C\overline{B}) + (D\overline{A}) + (\overline{BD})$
$(AB\overline{CD}) + (AC\overline{BD}) + (BC\overline{AD})$
$(AD) + (BD) + (AB\overline{C}) + (AC\overline{B}) + (BC\overline{A}) + (\overline{ABCD})$
$(AB\overline{C}) + (AC\overline{B}) + (BC\overline{A}) + (A\overline{BD})$

$C + \overline{A} + \overline{D}$

$(A\overline{D}) + (AC\overline{B}) + (BC\overline{A}) + (BD\overline{C})$

$(A\overline{C}) + (A\overline{D}) + (C\overline{A}) + (C\overline{D}) + (D\overline{A}) + (D\overline{C}) + (\overline{AB})$

$(AB\overline{C}) + (AB\overline{D}) + (A\overline{CD})$

$(AD\overline{B}) + (A\overline{CD}) + (BD\overline{AC})$

$(AD) + (BC) + (BD) + (A\overline{C})$

$(A\overline{B}) + (B\overline{D}) + (B\overline{AC})$

$(D\overline{A}) + (AB\overline{C}) + (A\overline{BD}) + (\overline{ABC})$

$(AB\overline{C}) + (AC\overline{D}) + (AD\overline{C}) + (BD\overline{C})$

$(AB\overline{C}) + (AC\overline{B}) + (BC\overline{A}) + (BD\overline{C}) + (\overline{BCD})$

$(B\overline{A}) + (B\overline{C}) + (B\overline{D}) + (D\overline{A}) + (D\overline{B}) + (D\overline{C}) + (\overline{AC}) + (AC\overline{B}) + (AC\overline{D})$

$(ABD\overline{C}) + (AC\overline{BD})$

$(A\overline{D}) + (AB\overline{C}) + (CD\overline{A})$

$(A\overline{D}) + (D\overline{A}) + (BC\overline{A})$

$(AB\overline{D}) + (AC\overline{B}) + (BD\overline{A}) + (CD\overline{B})$

$(BD) + (A\overline{B}) + (A\overline{C}) + (BC\overline{A}) + (\overline{BCD})$

$(A\overline{D}) + (AB\overline{C}) + (AC\overline{B}) + (BC\overline{A}) + (\overline{ABC})$

$\overline{B} + (A\overline{C}) + (A\overline{D}) + (C\overline{A}) + (C\overline{D}) + (D\overline{A}) + (D\overline{C})$

$(BCD\overline{A}) + (AC\overline{BD}) + (AD\overline{BC})$

$(AB\overline{C}) + (AC\overline{B}) + (BCD\overline{A})$

$(BD) + (ACD) + (BC\overline{A}) + (A\overline{CD})$

$(A\overline{D}) + (D\overline{B}) + (BC\overline{D}) + (D\overline{AC})$

$(AC\overline{B}) + (C\overline{AD}) + (AB\overline{CD})$

$(AB\overline{C}) + (AC\overline{B}) + (BC\overline{A})$

$(A\overline{B}) + (A\overline{C}) + (A\overline{D}) + (B\overline{A}) + (B\overline{C}) + (B\overline{D}) + (C\overline{A}) + (C\overline{B}) + (C\overline{D}) +$
$(D\overline{A}) + (D\overline{B}) + (D\overline{C})$

$(A\overline{D}) + (CD\overline{B}) + (D\overline{AC})$

$(AB\overline{C}) + (AB\overline{D}) + (A\overline{CD}) + (ACD\overline{B})$

$(C\overline{A}) + (D\overline{A}) + (B\overline{CD}) + (D\overline{BC})$

$(A\overline{B}) + (A\overline{C}) + (A\overline{D}) + (BD\overline{A}) + (BD\overline{C}) + (CD\overline{A}) + (CD\overline{B}) + (\overline{BCD})$

$(BCD) + (B\overline{CD}) + (AC\overline{BD})$

$A\overline{CD}$

$(BD\overline{A}) + (BD\overline{C}) + (ACD\overline{B}) + (\overline{ABC}) + (\overline{BCD})$

$(A\overline{D}) + (BC\overline{D})$

$(AC\overline{B}) + (BC\overline{A}) + (C\overline{AD}) + (AB\overline{CD})$

$(D\overline{A}) + (AB\overline{D}) + (AC\overline{D}) + (BC\overline{A})$

$(A\overline{D}) + (BCD) + (C\overline{BD})$

$(ACD\overline{B}) + (BCD\overline{A})$

$AD\overline{BC}$
$(D\overline{A}) + (BC\overline{A}) + (A\overline{BD}) + (A\overline{CD})$
$(AB\overline{C}) + (AB\overline{D}) + (A\overline{CD}) + (B\overline{CD}) + (ACD\overline{B}) + (BCD\overline{A})$
$\overline{A} + (B\overline{D}) + (C\overline{D}) + (D\overline{BC})$
$(B\overline{A}) + (B\overline{CD}) + (C\overline{BD}) + (AD\overline{BC})$
$A + B + C + \overline{D}$
$(A\overline{D}) + (AB\overline{C}) + (AC\overline{B})$
$(A\overline{B}) + (BC\overline{D}) + (BD\overline{A}) + (CD\overline{A})$
$(AB) + (ACD) + (BC\overline{D}) + (BD\overline{C}) + (CD\overline{B}) + (A\overline{CD}) + (D\overline{AB}) + (D\overline{AC})$
$(A\overline{D}) + (B\overline{C}) + (C\overline{B}) + (D\overline{A}) + (\overline{AB})$
$(A\overline{D}) + (B\overline{C}) + (D\overline{A}) + (CD\overline{B})$
$(AC\overline{B}) + (CD\overline{B}) + (A\overline{CD}) + (D\overline{AC})$
$(A\overline{B}) + (BC\overline{D}) + (BD\overline{A})$
$(A\overline{B}) + (C\overline{A}) + (B\overline{CD})$
$(A\overline{D}) + (B\overline{A}) + (B\overline{C}) + (\overline{AC}) + (AC\overline{B})$
$(AC) + (A\overline{D}) + (B\overline{A}) + (C\overline{D})$
$(AB) + (CD) + (A\overline{D}) + (B\overline{D}) + (D\overline{AB})$
$(AC\overline{B}) + (ABD\overline{C}) + (BCD\overline{A}) + (\overline{ABCD})$
$(A\overline{D}) + (BD\overline{AC})$
$(B\overline{A}) + (B\overline{C}) + (C\overline{D}) + (D\overline{C}) + (AD\overline{B})$
$A + (BCD) + (\overline{BCD})$
$(A\overline{D}) + (B\overline{D}) + (D\overline{AB}) + (D\overline{AC})$
$(AC\overline{D}) + (BD\overline{C}) + (A\overline{BD}) + (D\overline{AC})$
$(CD\overline{AB}) + (A\overline{BCD})$
$(A\overline{C}) + (D\overline{A}) + (D\overline{B}) + (BC\overline{D})$
$(B\overline{D}) + (C\overline{A}) + (D\overline{A}) + (D\overline{BC})$
$(B\overline{C}) + (B\overline{D}) + (D\overline{A}) + (AC\overline{B})$
$(AB\overline{C}) + (AC\overline{B}) + (AD\overline{B}) + (BC\overline{A}) + (\overline{ABD})$
$(B\overline{D}) + (C\overline{A}) + (D\overline{A}) + (A\overline{BC})$
$(A\overline{B}) + (B\overline{A}) + (B\overline{C}) + (\overline{CD})$
$(AB) + (AC) + (A\overline{D}) + (BC\overline{D}) + (D\overline{ABC})$
$(A\overline{D}) + (D\overline{A}) + (BC\overline{A}) + (D\overline{BC})$
$(AC\overline{B}) + (AD\overline{C}) + (BD\overline{A})$
$(A\overline{B}) + (A\overline{C}) + (D\overline{A}) + (\overline{BC}) + (BC\overline{A})$
$(C\overline{A}) + (D\overline{A}) + (D\overline{B}) + (A\overline{CD})$
$(ABD\overline{C}) + (BCD\overline{A}) + (A\overline{BCD})$
$B\overline{CD}$
$(B\overline{A}) + (AD\overline{B}) + (B\overline{CD}) + (C\overline{BD})$

$(A\overline{D}) + (BD\overline{A}) + (CD\overline{B})$
$(B\overline{A}) + (B\overline{C}) + (D\overline{B}) + (AC\overline{B})$
$(A\overline{D}) + (D\overline{A})$
$AD\overline{B}$
$(A\overline{D}) + (D\overline{A}) + (\overline{BC}) + (BC\overline{A})$
$(AB\overline{D}) + (AC\overline{B}) + (CD\overline{B}) + (BD\overline{AC})$
$(A\overline{D}) + (D\overline{A}) + (BC\overline{A}) + (BD\overline{C}) + (\overline{ABC})$
$(BC\overline{A}) + (ABD\overline{C}) + (ACD\overline{B})$
$\overline{A} + \overline{C} + \overline{D}$
$(AB\overline{D}) + (AC\overline{D}) + (AD\overline{BC})$
$(AB\overline{C}) + (BD\overline{C}) + (CD\overline{A}) + (A\overline{BD})$
$(AD\overline{B}) + (AD\overline{C})$
$A\overline{BCD}$
$(ABC\overline{D}) + (ABD\overline{C}) + (ACD\overline{B}) + (BCD\overline{A}) + (\overline{BCD})$
$(D\overline{A}) + (AC\overline{D}) + (BD\overline{C}) + (CD\overline{B}) + (A\overline{BD})$
$(AC\overline{B}) + (ABD\overline{C})$
$(AB\overline{C}) + (A\overline{CD}) + (ACD\overline{B})$
$(AC\overline{D}) + (BC\overline{A}) + (BD\overline{A}) + (CD\overline{A})$
$(A\overline{C}) + (A\overline{D}) + (C\overline{A}) + (C\overline{D}) + (D\overline{A}) + (D\overline{B}) + (D\overline{C})$
$(BD\overline{A}) + (CD\overline{A}) + (AC\overline{BD}) + (AD\overline{BC})$
$(A\overline{D}) + (B\overline{A}) + (B\overline{C}) + (D\overline{A}) + (\overline{AC})$
$A + B + D$
$(A\overline{D}) + (C\overline{D}) + (AC\overline{B}) + (BC\overline{A})$
$(ABC) + (ABD) + (ACD) + (BCD) + (\overline{BCD})$
$(AB\overline{D}) + (A\overline{CD}) + (ACD\overline{B}) + (BCD\overline{A})$
$(AB\overline{CD}) + (BD\overline{AC})$
$(D\overline{A}) + (BC\overline{D}) + (CD\overline{B}) + (A\overline{BD})$
$(CD\overline{B}) + (A\overline{CD})$
$(A\overline{B}) + (A\overline{C}) + (BC\overline{AD})$
$\overline{D} + (A\overline{B}) + (B\overline{A}) + (B\overline{C})$
$(AB\overline{C}) + (AC\overline{B})$
$(AD\overline{C}) + (BCD\overline{A})$
$(ACD\overline{B}) + (A\overline{BCD})$
$(AC\overline{B}) + (BCD\overline{A}) + (AB\overline{CD})$
$(CD\overline{B}) + (A\overline{CD}) + (BD\overline{AC})$
$(AB\overline{D}) + (AC\overline{B}) + (BD\overline{A})$
$C + \overline{A} + \overline{B} + \overline{D}$
$(AB\overline{C}) + (AD\overline{C}) + (BD\overline{A}) + (CD\overline{A})$

$\overline{A} + \overline{D} + (\overline{BC})$

$(AB\overline{D}) + (AC\overline{B}) + (BD\overline{C})$

$(A\overline{B}) + (A\overline{C}) + (D\overline{C}) + (\overline{BC}) + (BC\overline{A})$

$(AB\overline{C}) + (AC\overline{B}) + (AC\overline{D}) + (CD\overline{A}) + (B\overline{CD})$

$AB\overline{CD}$

$(BD\overline{A}) + (CD\overline{A}) + (AD\overline{BC})$

$(C\overline{BD}) + (B\overline{ACD}) + (D\overline{ABC})$

$(AB\overline{D}) + (AD\overline{BC})$

$B + (CD) + (A\overline{D}) + (D\overline{A})$

$(AC\overline{D}) + (AD\overline{B}) + (AD\overline{C}) + (BCD\overline{A})$

$B + (AD) + (\overline{CD})$

$(B\overline{CD}) + (AC\overline{BD})$

$(AB\overline{D}) + (C\overline{BD}) + (BCD\overline{A})$

$(AC\overline{B}) + (ABD\overline{C}) + (BCD\overline{A})$

$(A\overline{BD}) + (ABD\overline{C})$

$(A\overline{BD}) + (ABD\overline{C}) + (BCD\overline{A})$

$(ABC\overline{D}) + (ACD\overline{B}) + (BCD\overline{A}) + (A\overline{BCD})$

$(A\overline{D}) + (BC\overline{D}) + (BD\overline{C}) + (CD\overline{B})$

$AB\overline{D}$

$(A\overline{D}) + (B\overline{A}) + (C\overline{B}) + (D\overline{A})$

$(AB\overline{D}) + (AC\overline{B}) + (C\overline{BD}) + (BCD\overline{A})$

$(BC\overline{A}) + (A\overline{CD})$

$B + \overline{A} + \overline{C} + \overline{D}$

$(B\overline{A}) + (C\overline{BD}) + (D\overline{AC})$

$(A\overline{D}) + (B\overline{A}) + (B\overline{C}) + (D\overline{A}) + (CD\overline{B})$

$(D\overline{A}) + (D\overline{C}) + (AC\overline{BD})$

$(AB\overline{D}) + (AC\overline{B})$

$(AC\overline{B}) + (A\overline{BD}) + (ABD\overline{C})$

$(AB\overline{D}) + (AC\overline{D}) + (BD\overline{C}) + (CD\overline{B})$

$(A\overline{C}) + (C\overline{A}) + (D\overline{A}) + (D\overline{B}) + (\overline{AB})$

$B + (AC) + (CD) + (A\overline{D}) + (D\overline{A}) + (\overline{AC}) + (\overline{CD})$

$(B\overline{C}) + (AC\overline{B}) + (BD\overline{A}) + (A\overline{BD})$

$(A\overline{B}) + (B\overline{D}) + (D\overline{B}) + (D\overline{C})$

$(BC\overline{A}) + (BD\overline{C}) + (ACD\overline{B}) + (\overline{ABCD})$

$(AD\overline{B}) + (BC\overline{D}) + (A\overline{CD}) + (BD\overline{AC})$

$(AC\overline{B}) + (AC\overline{D}) + (A\overline{BD})$

$(C\overline{A}) + (C\overline{B}) + (C\overline{D}) + (D\overline{A}) + (D\overline{B}) + (D\overline{C}) + (AB\overline{C}) + (AB\overline{D})$

$(AC) + (A\overline{D}) + (BD\overline{A}) + (B\overline{AC}) + (C\overline{BD})$

$(ABD\overline{C}) + (ACD\overline{B}) + (BCD\overline{A}) + (\overline{ABCD})$
$(A\overline{B}) + (A\overline{C}) + (D\overline{A}) + (BC\overline{A})$
$(AB\overline{C}) + (AC\overline{B}) + (BD\overline{A})$
$(AB\overline{D}) + (AD\overline{B}) + (AD\overline{C}) + (BD\overline{A}) + (BD\overline{C}) + (CD\overline{A}) + (CD\overline{B})$
$AC\overline{B}$
$C\overline{BD}$
$(CD) + (D\overline{A}) + (A\overline{BD}) + (A\overline{CD})$
$(A\overline{D}) + (D\overline{A}) + (CD\overline{B}) + (\overline{ABC})$
$(A\overline{D}) + (D\overline{A}) + (BC\overline{A}) + (CD\overline{B})$
$(A\overline{B}) + (A\overline{D}) + (B\overline{A}) + (B\overline{D}) + (D\overline{A}) + (D\overline{B}) + (D\overline{C})$
$(A\overline{D}) + (B\overline{C}) + (C\overline{A}) + (D\overline{A})$
$(AB\overline{C}) + (AC\overline{B}) + (BC\overline{A}) + (\overline{ABCD})$
$(A\overline{B}) + (A\overline{C}) + (B\overline{AD})$
$(AB\overline{C}) + (AC\overline{B}) + (AD\overline{B})$
$(A\overline{D}) + (AC\overline{B}) + (C\overline{BD}) + (BCD\overline{A})$
$(\overline{AB}) + (CD\overline{B}) + (A\overline{CD}) + (D\overline{AC})$
$(B\overline{A}) + (C\overline{A}) + (B\overline{CD}) + (AD\overline{BC})$
$(AB\overline{C}) + (AC\overline{B}) + (A\overline{BD}) + (B\overline{CD}) + (BCD\overline{A})$
$(AB\overline{C}) + (AB\overline{D}) + (ACD\overline{B}) + (BCD\overline{A})$
$(D\overline{A}) + (AC\overline{D}) + (BD\overline{C}) + (CD\overline{B}) + (A\overline{BD}) + (B\overline{AC})$
$C + (A\overline{D}) + (D\overline{A}) + (\overline{AB})$
$(A\overline{D}) + (B\overline{A}) + (B\overline{D}) + (D\overline{A}) + (\overline{AC}) + (\overline{CD}) + (AC\overline{B}) + (CD\overline{B})$
$(AC\overline{B}) + (AD\overline{C})$
$(C\overline{D}) + (D\overline{C}) + (AB\overline{C}) + (AD\overline{B}) + (BD\overline{A})$
$(A\overline{B}) + (A\overline{D}) + (B\overline{A}) + (B\overline{D}) + (D\overline{A}) + (D\overline{B}) + (\overline{AC})$
$(A\overline{BC}) + (A\overline{BD}) + (A\overline{CD}) + (BC\overline{AD})$
$(AC\overline{D}) + (B\overline{AC}) + (C\overline{AB}) + (D\overline{AB})$
$A + B + \overline{C} + \overline{D}$
$(AD\overline{C}) + (BD\overline{A}) + (CD\overline{A}) + (AC\overline{BD})$
$(AB) + (AC) + (BC) + (BD) + (CD) + (A\overline{D}) + (D\overline{A}) + (\overline{ABC}) + (\overline{BCD})$
$(B\overline{A}) + (D\overline{A}) + (C\overline{BD})$
$(CD\overline{A}) + (AC\overline{BD}) + (AD\overline{BC})$
